# Role of Sensory Experience in Functional Development of *Drosophila* Motor Circuits

**DOI:** 10.1371/journal.pone.0062199

**Published:** 2013-04-19

**Authors:** Akira Fushiki, Hiroshi Kohsaka, Akinao Nose

**Affiliations:** 1 Department of Complexity Science and Engineering, Graduate School of Frontier Sciences, University of Tokyo, Kashiwa, Chiba, Japan; 2 Department of Physics, Graduate School of Science, University of Tokyo, Bunkyo-ku, Tokyo, Japan; Columbia University, United States of America

## Abstract

Neuronal circuits are formed according to a genetically predetermined program and then reconstructed in an experience-dependent manner. While the existence of experience-dependent plasticity has been demonstrated for the visual and other sensory systems, it remains unknown whether this is also the case for motor systems. Here we examined the effects of eliminating sensory inputs on the development of peristaltic movements in *Drosophila* embryos and larvae. The peristalsis is initially slow and uncoordinated, but gradually develops into a mature pattern during late embryonic stages. We tested whether inhibiting the transmission of specific sensory neurons during this period would have lasting effects on the properties of the sensorimotor circuits. We applied Shibire-mediated inhibition for six hours during embryonic development (15–21 h after egg laying [AEL]) and studied its effects on peristalsis in the mature second- and third-instar larvae. We found that inhibition of chordotonal organs, but not multidendritic neurons, led to a lasting decrease in the speed of larval locomotion. To narrow down the sensitive period, we applied shorter inhibition at various embryonic and larval stages and found that two-hour inhibition during 16–20 h AEL, but not at earlier or later stages, was sufficient to cause the effect. These results suggest that neural activity mediated by specific sensory neurons is involved in the maturation of sensorimotor circuits in *Drosophila* and that there is a critical period for this plastic change. Consistent with a role of chordotonal neurons in sensory feedback, these neurons were activated during larval peristalsis and acute inhibition of their activity decreased the speed of larval locomotion.

## Introduction

Neural control of almost all rhythmic behaviors, such as walking, chewing and swimming, are thought to be generated by neural circuits called central pattern generators (CPGs). CPGs are networks of neurons that produce rhythmic motor outputs without depending on sensory inputs [1], [2]. From the beginning of the last century, CPGs have been found in many kinds of animals and are shown to play critical roles in motor generation.

During movement, sensory signals from muscles and other body regions often alter the pattern of CPG activity. For example, in the walking stick insect *Carausius morosus*, sensory information from leg proprioceptors is utilized to modify the strength of the muscle contractions [3]. Sensory feedback may help to generate a functional locomotive pattern by adapting an animal’s movements to its environmental demands. Although sensory feedback is not necessary for generating the rhythms, it may therefore play an important role in shaping the motor patterns.

Does sensory feedback also play a role in the development of the motor system? Sensory experience plays critical roles in the development of the sensory systems [4]. Genetic programs can form rough patterns in sensory circuits in the absence of neural activity, but the final tuning and refinement of the circuits depends on neural activity induced by sensory experience. In many systems, there is a critical period, a strict time window during which sensory experience is particularly important for the fine-tuning of the circuits. A well-known example is the development of mammalian binocular vision [5]. Since sensory systems monitor the output of motor circuits and feed the information back to the central circuits, sensory feedback may well be important for activity-dependent refinement of the motor system. However, there is little information about sensory contributions to the development of the motor system.

*Drosophila* larval locomotion is an attractive model to investigate the role of sensory experience in the development of motor circuits [6]. In addition to the availability of powerful tools for genetic manipulation in this species, *Drosophila* larval locomotion is a simple behavior based on a repetitive pattern that can be easily quantified. Forward larval locomotion is accomplished through propagation of muscle contractions from the posterior to anterior of the body. Several studies have demonstrated that sensory feedback is a critical component for normal locomotion [7], [8]. The peripheral nervous system (PNS) in *Drosophila* larvae comprises distinct cell types, each with characteristic dendritic projections. These include external sensory (es) neurons and chordotonal organs (chos), which terminate in a single ciliated dendrite, as well as multiple dendritic (md) neurons, which project multiple dendrites [9]. Multiple dendritic neurons are further classified, based on the complexity of their dendritic arbors, into bipolar dendritic (bd) neurons and four classes of dendritic arborization (da) neurons (I–IV) [10]. Among these, bd and class-I da neurons, which have relatively simple dendritic arbors, appear to be particularly important for normal larval locomotion (hereafter, we refer to these neurons as md neurons for simplicity) [8], [11], [12]. When function of the md neurons is temporally inhibited, the speed of locomotion is greatly reduced. Thus, it has been proposed that these neurons function as proprioceptors that feed back the status of the muscle contraction, and this feedback information is critical for fast wave propagation. While temporal inhibition of other sensory neurons does not cause strong crawling defects, some of these neurons may play minor roles in sensory regulation of larval locomotion [8], [12]. In mutant flies with no or defective chos, the duration of linear locomotion is decreased and frequency of turning is increased [13]. In these mutants, the speed of locomotion is also reduced, although the reduction is much smaller than what is seen when the function of the md neurons is inhibited.

Initial peristaltic movement is seen in developing embryos several hours before hatching [14], [15]. At ∼4 hours before hatching, episodic bursts of uncoordinated muscle movements, which are driven by the developing neural circuits, first appear. The movements are initially asynchronous across all segments, and then they gradually develop into complete waves that travel sequentially along the entire body of the embryo. The first complete wave, which occurs ∼3 h before hatching, is slow (propagation duration = ∼10 sec) and uncoordinated, and then it gradually develops to a mature pattern during late embryonic and early larval stages. The final pattern of peristalsis (propagation duration = ∼1 sec) is completed within several hours after hatching, and the speed of peristalsis stays the same throughout the larval period. These observations suggest that the motor circuits mature while the animals are performing premature locomotive motion.

In this study, we tested whether the sensory experience of the muscle movements is required for proper development of motor circuits. To do this, we inhibited the function of putative sensory feedback neurons, md neurons or chos, during embryonic development and examined whether the manipulation elicited any change in the properties of the mature circuits. We found that inhibition of chos but not md neurons caused a long-lasting decrease in the speed of larval locomotion. Furthermore, there was a critical time window in which the activity of chos was required for proper development of the motor circuits. These results provide evidence for a role of sensory experience in the development of motor circuits.

## Materials and Methods

### Fly Strains

The following fly strains were used: *iav(inactive)-GAL4* [16], *109(2)80-GAL4* [8], *UAS-GCaMP3* [17], *UAS-Shibire^ts^* [18], *UAS-TeTxLc* [19] and *iav* ^1^ [20]. Flies were raised on conventional cornmeal agar medium at 25°C.

### Behavioral Analysis

**Figure 1:**
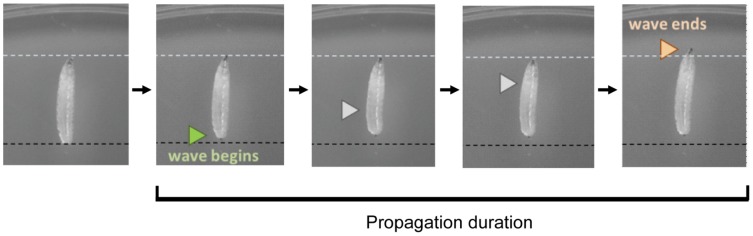
Behavioral analysis (locomotion analysis). To measure larval propagation duration, we videotaped the animals’ behavior using a CCD camera (30 frames/sec for 30 seconds). We manually calculated the duration of waves (10 waves per larva) using ImageJ software.

Second- and third-instar larvae were gently washed in deionized water and then placed on an apple juice agar plate. After acclimation (5 min), we measured the duration of peristalsis (elapsed time between the landing of the posterior end and elongation of the head, also called propagation duration, Figure 1). The movements of the larvae were videotaped under a microscope (SZX16, Olympus, Japan) using a XCD-V60 CCD camera (30 frames/sec for 30 seconds) and the movies were downloaded into VFS-42 (Vision Freezer, Chori imaging). We manually calculated the propagation duration (10 waves per larva) using ImageJ 1.44 software.

### Temperature Shift Experiments

Parental flies were reared in an egg collection cup with an agar plate at 25°C. Eggs were laid for 1 h on an agar plate containing yeast paste. ∼30 embryos were then transferred to a new plate without yeast paste. For the conditional inhibition assay using *Shibire^ts^*, the plates were held at a restrictive temperature (RT, 32°C) on a heat plate (Thermo Plate, Tokai Hit, Japan) during the desired developmental periods. After the conditional inhibition, the embryos/larvae were transferred to a new plate with yeast paste and reared at the permissive temperature (PT, 25°C).

### Calcium Imaging

Wandering third-instar larvae were selected, gently washed, and pinned on a sylgard-coated dish. Larvae were dissected in Ca^2+^-free normal saline (NaCl 140 mM, KCl 2 mM, MgCl_2_ 6 mM, Hepes-NaOH 5 mM, Sucrose 36 mM (pH 7.1)). The internal organs were removed without scratching the ventral nerve cord (VNC) and axons. After rinsing the sample with Ca^2+^-free normal saline, the buffer was replaced with 2 mM Ca^2+^ Ringer solution (NaCl 130 mM, KCl 5 mM, MgCl_2_ 2 mM, CaCl_2_ 2 mM, Hepes-NaOH 5 mM, Sucrose 36 mM (pH 7.3)). To fix the position of the VNC, a pin was placed between the brain and the mouth hook. Imaging was performed on a fluorescence microscope (MVX10, Olympus, Japan) equipped with a CCD camera (XCD-V60, Sony, Japan). The images were acquired and downloaded into VFS-42 (Vision Freezer, Chori imaging) at 30 frames/sec, 640×480 pixels. For image analyses, ImageJ was used. The pseudocolored images were made using RGB color to observe the autofluorescence of muscle contractions.

### Immunocytochemistry

Dissected larvae were fixed in phosphate buffered saline (PBS, NaCl 137 mM, KCl 2.7 mM, Na_2_HPO_4_ 8.1 mM, KH_2_PO_4_ 1.5 mM (pH 7.3)) containing 4% paraformaldehyde for 30 min at room temperature. After two 15 min washes with 0.2% Triton X-100 in PBS (PBT), the larvae were incubated with 5% normal goat serum in PBT for 30 min. The larvae were then incubated overnight at 4°C with the primary antibody (rabbit anti-GFP, A2020, Frontier Institute, 1∶1000). After two 15 min washes, the larvae were incubated overnight at 4°C with the secondary antibody (Alexa Fluor 488-conjugated goat anti-rabbit IgG, A11034, Invitrogen, 1∶300). Images were acquired using a confocal microscope (FV1000, Olympus, Japan).

### Statistical Analysis

We analyzed the data using Welch’s *t* test, Mann-Whitney *U* test, and one-way analysis of variance (ANOVA) followed by Tukey’s or Dunnett’s tests for multiple comparisons. Statistical significance is denoted by asterisks: ***P<0.001; **P<0.01; *P<0.05. All statistical tests were performed using R-project software (http://www.r-project.org). The results are stated as mean ± s.d., unless otherwise noted.

## Results

### Temporal Inhibition of Chordotonal Organs at Late Embryonic Periods Alters the Properties of Larval Motor Circuits

Initial embryonic motor events start at stage 16 (∼15 h AEL) as brief, myogenic muscle twitches. Nerve-driven muscle contraction initiates at ∼17 h AEL as episodes of uncoordinated bursting activity. Shortly thereafter, partial waves that involve multiple segments appear, followed by the appearance of complete waves. The waves are initially slow (∼10 sec per wave) and uncoordinated, but gradually speed up to become a mature pattern (∼1 sec per wave) several hours after hatching [15]. Thus, the motor circuits likely develop into mature circuits during these late embryonic periods.

Given that neural circuits are often plastic and highly susceptible to environmental changes during their formation, we reasoned that inhibiting the activity of sensory input in late embryonic periods may change the properties of the circuits. We therefore examined whether temporal inhibition of putative sensory feedback neurons, md neurons and chos, has a lasting effect on the properties of the motor system. As a quantitative measure of the properties of motor circuits, we used propagation duration of peristalsis, which provides an indirect measure of the locomotion speed. The speed of locomotion increases during the maturation period of the motor circuits but remains stable when the circuits mature in the late first-instar larval stage. Furthermore, the speed is reasonably constant among different genotypes. (Note, however, that the speed varies at different temperatures: larvae crawl faster at higher temperatures (e.g., ∼30°C) compared to room temperature). We applied Shibire-mediated neural inhibition for six hours at late embryonic stages (15–21 h AEL) and analyzed the effects on larval locomotion at the second- or third-instar larval stage. The *Shibire* transgene (*UAS- Shibire^ts^*) blocks synaptic vesicle recycling and disrupts synaptic transmission at restrictive temperatures (>29°C) [18]. Induction and reversal occurs within a few minutes after the temperature shift between the permissive temperature (PT) and the restrictive temperature (RT). To express *Shibire^ts^* in md neurons or chos, we used GAL4 drivers, *109(2)80-GAL4* and *iav-GAL4* respectively. The driver *109(2)80-GAL4* is expressed in all the md neurons and in small subsets of cells in the CNS [8]. A previous study has shown that a maximum level of inhibition of md neurons can be achieved with *109(2)80-GAL4,* suggesting that this GAL4 line drives expression in all or most of the md sensory feedback neurons. The driver *iav-GAL4* is expressed only in chos: no detectable expression is seen in other sensory neurons or cells in the CNS.

**Figure 2:**
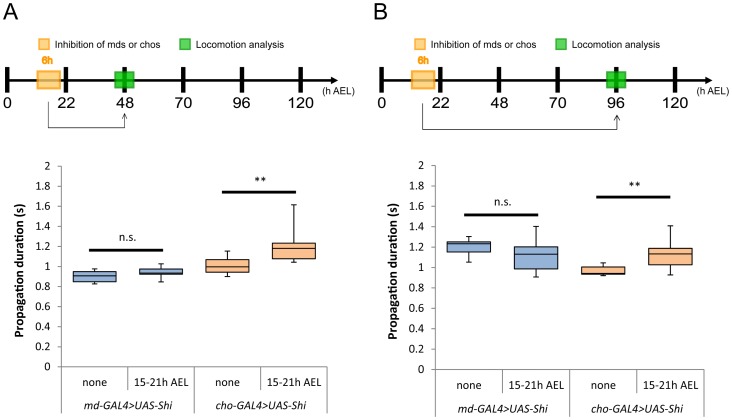
Inhibition of chos at late embryonic stage affects peristalsis in mature larvae. Effects of inhibiting the activity of multidendritic neurons (md neurons, mds) or chordotonal organs (chos) during 15–21 h AEL. Inhibition of chos (orange boxes) but not md neurons (blue boxes) led to propagation defects in the second (A) and third (B) instar. Box plots indicate the median value (horizontal line inside the box), 25–75% quartiles (box), and the data range (whiskers). Statistical significance was determined by Mann-Whitney *U* test (**P<0.01; n.s., not significant). For all conditions, n = 10. RT, restrictive temperature. *109(2)80-GAL4* and *iav-GAL4* were used as md neurons and chos drivers, respectively.

We observed a significant increase in propagation duration when the Shibire-mediated inhibition was applied to chos (Figure 2A; 1.22±0.19 sec compared to 1.00±0.09 in the control larvae, which have the same genotype but did not undergo the temperature shift; p<0.01). Slower locomotion was seen at the second- and third- instar larval stage, indicating that the plastic change can last more than three days (Figure 2B; 1.13±0.14 sec compared to 0.97±0.05 in the control larvae; p<0.01). These results suggest that temporal inhibition of chos during late embryonic periods has lasting effects on the properties of the motor circuits. In contrast, inhibition of md neurons caused no alteration in the speed of larval locomotion (Figure 2A and B; propagation duration, 0.94±0.05 sec compared to 0.90±0.06 in the control larvae [analysed at 48 h AEL], 1.11±0.15 sec compared to 1.20±0.08 in the control larvae [analysed at 96 h AEL]; p>0.05). These results suggest that the sensory experience mediated by chos, but not by md neurons, at late embryonic periods is required for proper maturation of the motor circuits.

### The Role of Chordotonal Organs in the Regulation of Mature Motor Circuits

**Figure 3:**
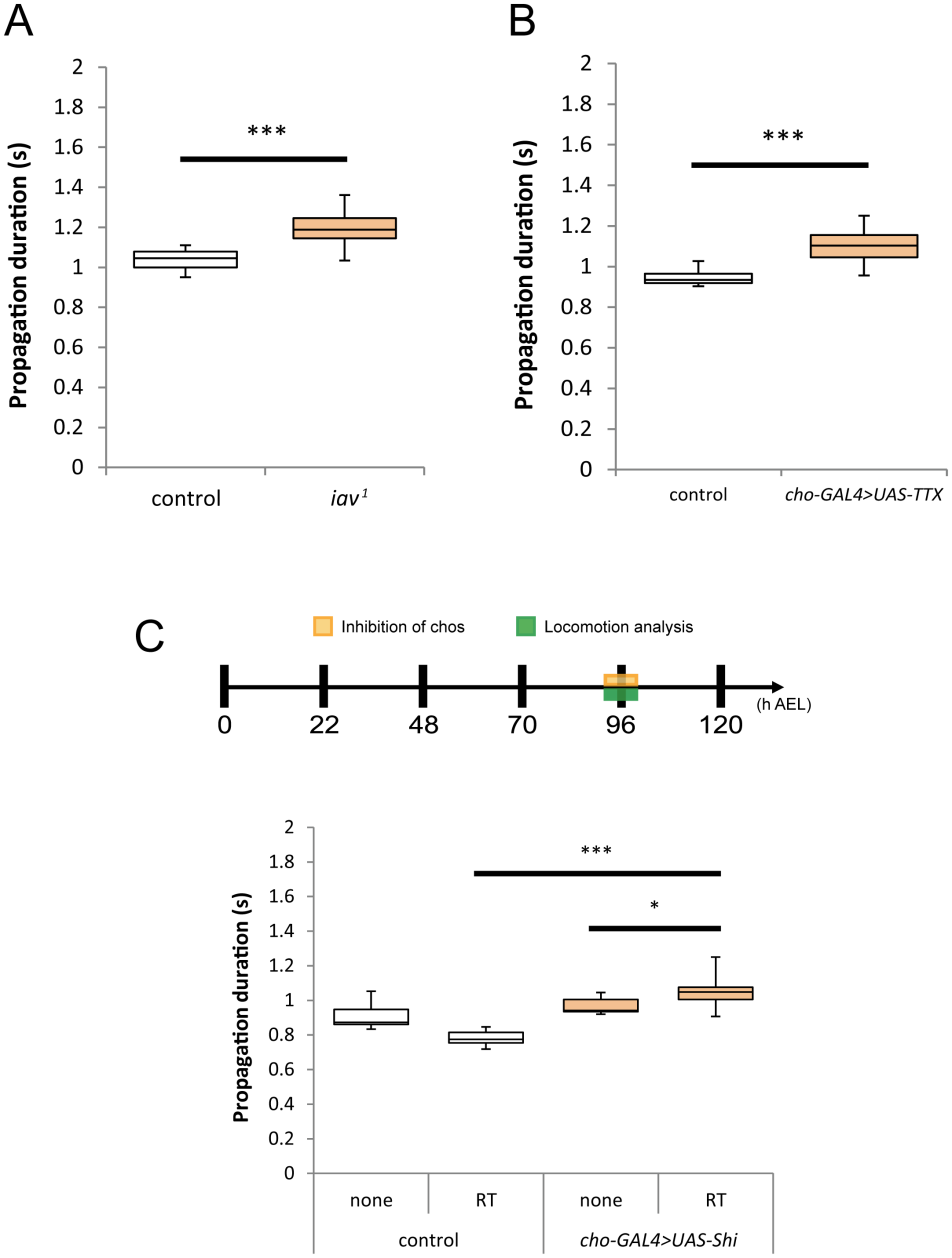
Chordotonal organs regulate locomotion through sensory feedback to the CNS. (A, B) Increase in propagation duration in *iav^1^* mutants (A) and in larvae expressing *TeTxLc* (*TTX*) in chos (B). (C) Acute inhibition of chos with *Shibire^ts^* increased the propagation duration of the larvae. Statistical analysis was done by Welch’s *t* test (***P<0.001; *P<0.05; n.s., not significant). For all conditions, n = 10. RT, restrictive temperature. Controls were as follows: *yw* (A), *yw>UAS-TTX* (B), *yw>UAS-Shi* (C).

The observed effects of chos inhibition on larval locomotion suggest that these neurons convey some sensory information to the motor circuits in the embryos and that sensory inputs are important for proper development of the motor circuits. This observation prompted us to reexamine the role of chos in larval locomotion. Previous studies showed that mutant larvae that have no or defective chos show some defects in locomotion (see Introduction). However, the precise role of chos in larval locomotion has not been examined. In particular, the effects of temporal inhibition of chos activity have not been analyzed in a quantitative manner. Using propagation duration as a quantitative measure, we first confirmed previous reports showing that dysfunction of chos results in malfunction of motor abilities [13]. In third instar larvae mutant for the *inactive* (*iav*) gene, which encodes a transient receptor potential vanilloid (TRPV) channel that is essential for neuronal function, there was a significant increase in the propagation duration (Figure 3A). A similar increase in the propagation duration was observed when the function of chos was inhibited by expression of the tetanus toxin light chain (*UAS-TeTxLc*), which irreversibly cleaves synaptobrevin and disrupts chemical synaptic transmission (Figure 3B) [19]. Finally, we studied the effects of acute inhibition of chos by expressing *Shibire^ts^* in these neurons. When the function of chos was temporally inhibited with *Shibire^ts^*, there was a significant increase in the propagation duration, compared to the control larvae at the same temperature (32°C) (Figure 3C; 1.06±0.09 sec in *iav-Gal4/UAS-Shibire^ts^* compared to 0.78±0.04 in the control [*+/UAS-Shibire^ts^*]; p<0.001). The effect was not as dramatic, however, as when the function of md neurons is inhibited ([8]; our own observation). As another control, we also compared propagation duration of *iav-Gal4/UAS-Shibire^ts^* larvae at a PT (25°C) to that at a RT (32°C). Even though the speed of larval locomotion is normally higher at restrictive temperatures (compare propagation at PT and RT of control larvae in Figure 3C), larvae expressing *Shibire^ts^* crawl more slowly at RTs than at PTs (propagation duration, 1.06±0.09 sec at 32°C compared to 0.97±0.05 at 25°C; p<0.05). Thus, the speed of larval locomotion decreases when the function of chos is compromised. These results suggest that chos send sensory feedback to the CNS, which contributes to an increase in the speed of locomotion.

### Calcium Imaging of Chordotonal Organs during Peristaltic Muscle Contractions

**Figure 4:**
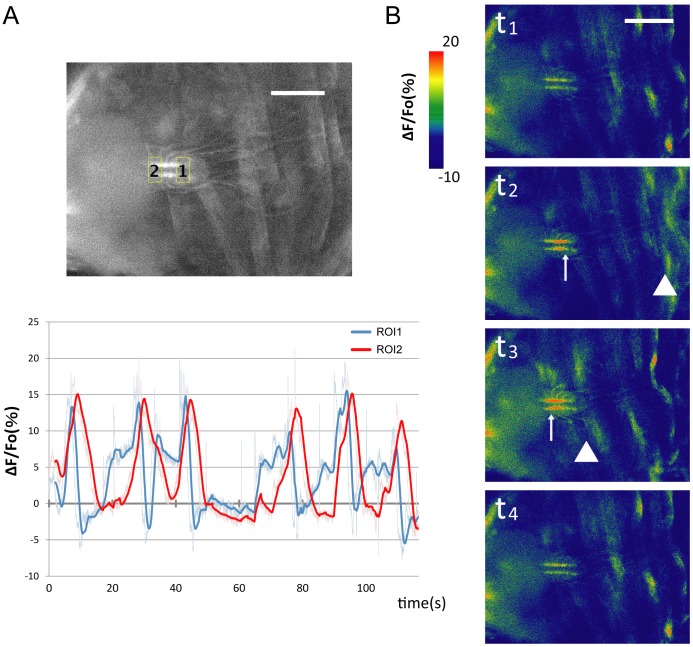
Calcium imaging in chordotonal organs during muscle contractions. GCaMP-based calcium imaging in chos. (A) Representative fluorescence change (ΔF/F_0_) of GCaMP3 in the axon terminals of chos in a posterior (ROI1, blue) and an anterior (ROI2, red) region of the CNS were plotted (bottom). Note that the signal rise in the posterior region precedes that in the anterior region in consecutive rounds of activity propagation. The amplitude of calcium signals was smoothed (moving average of 60 points). The top panel shows the position of the ROIs. (B) Time-lapse images of the fluorescent intensities (t_1_ to t_4_). Anterior is to the left and posterior is to the right. Arrows denote the signal rise in chos terminals in the ventral nerve cord. Arrowheads denote positions of muscle contractions, which were detected using the autofluorescence images of muscles. Note that the activation of chos and segmental muscle contraction propagate at a similar timing (see Movie S1). Scale bars represent 250 µm.

Chos are internal sensory organs that are thought to function as proprioceptors and/or mechanoreceptors [21]. They may therefore detect changes in the position or tension of the body wall muscles during peristalsis, and then relay the information to the CNS. If this is the case, chos should be activated in a manner correlated with peristaltic motion. To pursue this idea, we conducted calcium imaging of chos. We expressed a genetically coded calcium sensor (*GCaMP3*) [17] in chos and performed calcium imaging in dissected larvae undergoing peristalsis. We focused on the signal change in the axon terminals of chos in the ventral nerve cord, which extend along a longitudinal tract in the ventral neuropile [9]. We observed strong calcium signals in each neuromere, which propagates along the AP axis concomitant with the propagation of muscular contraction (Figure 4 and Movie S1). Thus, chos are activated during peristalsis with a similar timing as the contraction of the body-wall muscles. This observation is consistent with the idea that chos convey sensory-feedback information about muscular contraction. We also detected sporadic activities of chos, which did not coincide with motor activity. The signal level of such spontaneous activity was much lower than the level of activity seen during peristalsis.

### Existence of a Critical Period for Plastic Change Induced by Sensory Inhibition

As described above, inhibition of chos during a six-hour period (15–21 h AEL) in late embryonic development had a lasting effect on the crawling speed of the larvae. We next asked whether inhibition of chos for a shorter period also alters larval locomotion and, if so, whether there is a developmental time window in which the animal are particularly sensitive to sensory manipulation. We also asked whether similar inhibition of chos in larval stages induces the plastic change in larval behavior. To do this, we applied Shibire-mediated inhibition for two hours in different embryonic and larval time windows and analyzed the animals for locomotion defects at later stages.

**Figure 5:**
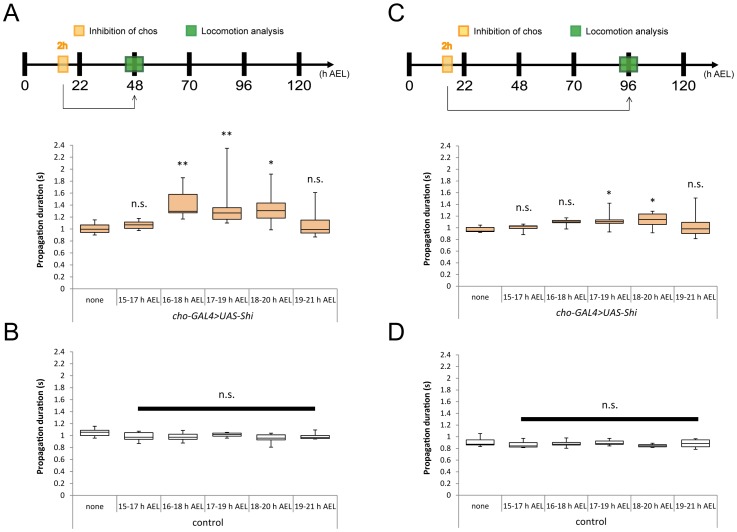
A critical period for the maturation of the sensorimotor circuit. (A, C) The effects of temporal inhibition of chos neurons at various embryonic stages on larval locomotion, analyzed at the second (A, 48 h AEL) and third instar (C, 96 h AEL) stage. A two-hour temperature shift to a restricted temperature was applied to *iav-GAL4*>*UAS-Shi^ts^* embryos at various stages during embryogenesis. The temperature shift at specific embryonic periods increased the propagation duration. (B, D) The same temperature shift applied to control embryos (*yw*>*UAS-Shi^ts^* ) had no effect on locomotion, when analyzed at the second (B) and third instar (D) stage. Statistical significance was determined by one-way ANOVA followed by Dunnett’s test for multiple comparisons (**P<0.01; *P<0.05; n.s., not significant compared with *iav-GAL4*>*UAS-Shi^ts^* (none) control). For all conditions, n = 10. RT, restrictive temperature.

We first induced 2-h inhibition periods in the embryos at different time windows between 15 and 21 h AEL and examined larval locomotion in second- and third- instar larval stages (48 h and 96 h AEL, respectively) (Figure 5). Analysis of the locomotion in second instar larvae showed that the two-hour inhibition applied between 16 and 20 h AEL significantly increased the propagation duration (Figure 5A; 1.40±0.23 sec [16–18 h AEL temperature shift], 1.36±0.36 sec [17–19 h AEL] and 1.35±0.29 sec [18–20 h AEL] compared to 1.00±0.09 in the control larvae without the temperature shift; p<0.01). No such increase in propagation duration was seen when inhibition was applied at an earlier or later stage (Figure 5A; 1.07±0.07 sec [15–17 h AEL temperature shift] and 1.10±0.26 sec [19–21 h AEL]; p>0.05). When the same two-hour temperature shift was applied to control larvae, there was no change in the propagation duration, indicating that a temperature shift in these embryonic periods itself has no effect on the development of motor behavior (Figure 5B; 0.98±0.06 sec [16–18 h AEL temperature shift], 1.01±0.03 sec [17–19 h AEL] and 0.96±0.07 sec [18–20 h AEL] compared to 1.05±0.07 sec in the control larvae without the temperature shift; p>0.05). The degree of the increase in the propagation duration was similar to that seen when six-hour inhibition was applied (p>0.05, data not shown). These results show that inhibition of chos activity for two hours is sufficient to change properties of the motor circuits and there is a critical period for when the circuits are particularly sensitive to the inhibition. Similar effects were seen when the locomotion assay was performed at the third-instar larval stage (Figure 5C and D; 1.10±0.06 sec [16–18 h AEL temperature shift], 1.11±0.13 sec [17–19 h AEL] and 1.13±0.13 sec [18–20 h AEL] compared to 0.97±0.05 in the control larvae without the temperature shift; p<0.05). Thus, the effects of the two-hour inhibition in the embryos were long lasting. Note, however, that the effects observed in third instar larvae were less dramatic than those observed in the second instar larvae, suggesting that the defects were partially restored during the larval life.

**Figure 6:**
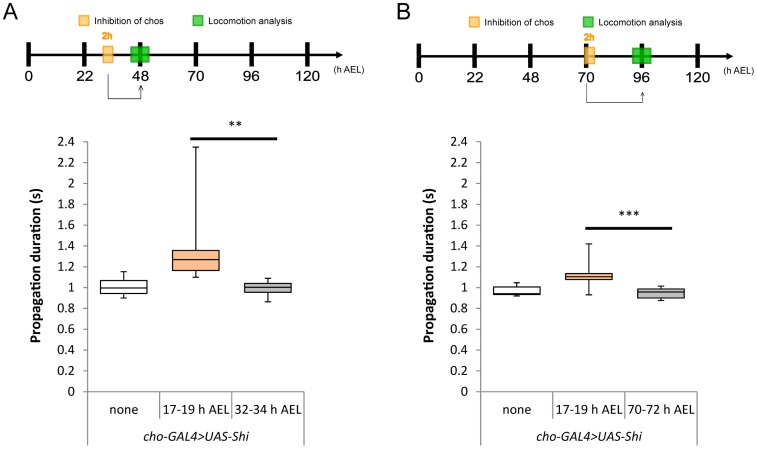
Inhibition of chordotonal organs at larval stage has no effect on the speed of larval locomotion. In contrast to the two-hour inhibition during the embryonic stage (17–19 h AEL), those during first (A, 32–34 h AEL) and third (B, 70–72 h AEL) instar stage had no effect on the speed of locomotion. Analyzed at second (A, 48 h AEL) and third instar (B, 96 h AEL) stages. Statistical significance was determined by one-way ANOVA followed by Tukey’s test for multiple comparisons (***P<0.001; **P<0.01). For all conditions, n = 10. RT, restrictive temperature.

We next studied whether plastic change can occur when chos are inhibited during larval periods. Two-hour temperature shifts were applied at two different stages of larval development, 32–34 h and 70–72 h AEL, and the effects on locomotion were analyzed at 48 h and 96 h AEL, respectively. No alteration in the speed of locomotion was observed after these manipulations (Figure 6A and B) (0.99±0.07 sec [32–34 h AEL temperature shift], 0.95±0.05 sec [70–72 h AEL] compared to 1.00±0.09, 0.97±0.05 in the control larvae without the temperature shift; p>0.05). These results suggest that the plastic change in motor circuits can only be induced when the sensory inhibition occurs during specific periods of late embryonic development.

## Discussion

### Role of Sensory Feedback in the Maturation of Motor Circuits

Animals modify the activity of locomotor outputs when their surroundings change, which is an efficient strategy that enables them to react appropriately to a wide variety of possible environmental situations. Modifications are mediated by sensory feedback about the current status of the locomotor organs and their interaction with the environment; this information is transmitted to the central nervous system. While the role of sensory feedback in the modulation of the mature motor circuits is well established, it has been unclear whether sensory feedback also plays a role during the construction of the motor circuits during development. In this study, we found that the activity of chos during embryonic development is required for proper formation of motor circuits. Furthermore, there was a critical developmental period during which this activity-dependent modulation can occur.

We inhibited neural transmission mediated by specific sensory neurons, md neurons and chos, during late embryonic periods to determine whether sensory information plays a role in the development of motor circuits in *Drosophila*. Even though md neurons play a major role in the regulation of locomotion speed in third instar larvae, inhibition of these neurons in the embryonic period did not cause detectable changes in larval locomotion. In contrast, we found a modest but reproducible alteration in the properties of the mature motor circuits when we temporally inhibited the neural transmission of chos. This observed cell-type specificity suggests that it is not the general lack of sensory feedback but rather inputs conveyed by specific sensory pathways that are important for proper development of motor circuits. However, we cannot completely exclude the possibility that md neurons might also be important for motor circuit development because the GAL4 line used in this study (*109(2)80-GAL4*) drives expression not only in class I md neurons but also in all other md neurons (class II, III and IV) and in some CNS interneurons. It is possible that the effects of inhibition of class I during a critical period may have been masked by the opposite effects caused by inhibition of some other GAL4-positive neurons.

Here we blocked the neural transmission of chos with *Shibire^ts^* during the embryonic period when motor circuits mature (15–21 h AEL) to realize a mature speed of locomotion. We found that the temporal inhibition perturbed maturation and caused an enduring effect on the speed of larval locomotion that lasts until the second or third instar larval stages. Further narrowing of the temporal window revealed that two-hour inhibitions during 16–20 h AEL, but not those during earlier or later time periods, are sufficient to cause the plastic change. A concern using *Shibire^ts^* for temporal inhibition is possible continuation of the effect after the temperature shift back to a permissive temperature [22]. The lasting effects we observed therefore could simply be due to an enduring effect of *Shibire^ts^* in the larvae. However, the cell-type and temporal specificity we observed argues against this possibility and points to a specific role of chos-mediated neural transmission in the maturation of motor circuits.

### Role of Chordotonal Organs in Sensory Feedback

The effect of chos inhibition on the maturation of motor circuits prompted us to reevaluate the role of chos in the regulation of larval locomotion. The role of chos in the regulation of locomotion has been controversial. While several previous studies have suggested a role for chordotonal mutants in locomotion, other studies report that depriving chos-mediated transmission had no apparent effect on larval locomotion [8], [12], [13], [16]. However, these previous studies largely relied on qualitative analyses of the behavior of the larvae. We therefore used quantitative analyses of the change in speed of locomotion to investigate the role of chos in larval locomotion. We first confirmed previous observations that the speed of larval locomotion decreases in *inactive* mutants [16], [20]. Since this gene is expressed exclusively in chos, the locomotion defect is probably caused by the malfunction of these neurons. We also observed a similar decrease in the speed of locomotion when we blocked the function of chos with *TeTxLc*. Finally, we found that acute inhibition of chos with *Shibire^ts^* decreased the speed of locomotion. The effect of inhibiting chos was not as dramatic as when the function of md neurons was compromised. However, these results suggest that chos contribute in a minor manner to the regulation of speed of larval locomotion. Our calcium imaging analyses further supported this notion. We observed that chos are activated along the segments coincidentally with the wave of muscular contraction. This is consistent with the idea that chos sense and are activated by the mechanical force generated by muscle contraction and convey the information to the CNS. Since the speed of locomotion is decreased upon inhibition of chos, sensory feedback likely contributes to fast propagation of motor activities in the CNS, as has been proposed for md neurons [8]. A recent study showed a role for larval chos in the sensation of vibration [23]. It could be that chos are multimodal, sensing and conveying multiple stimuli, as has been proposed for other sensory neurons in *Drosophila*.

### Critical Periods for Activity-dependent Maturation of Motor Circuits

There are well-established critical periods for activity-dependent maturation or reorganization of neural circuits during the development of the sensory system [24], [25]. Well-investigated examples include binocular vision in mammals and song learning in birds [5], [26]. Recent studies have shown that experience-dependent modifications of the sensory system also occur in *Drosophila*, whose nervous system was generally believed to be hard-wired [27], [28]. In the *Drosophila* larval visual system, changes in light experience induce homeostatic alterations in the functional and structural properties of an interneuron population in the circuits [27]. Similarly, excessive exposure to CO_2_ induces structural and functional changes in interneurons in the olfactory circuits in adults [28]. In the latter case, the plastic change has been reported to occur only within a critical time window. However, whether such experience- or activity-dependent modifications of neural circuits occur in the motor system and whether there is a critical period for the plastic changes have not been well investigated in any organisms. In this study, we showed that neural activity mediated by putative sensory-feedback neurons is required for proper maturation of the motor circuits in *Drosophila* larvae during a specific developmental period.

While our study was in progress, Crisp et al. (2011) reported that blocking neuronal transmission or inducing abnormal patterns of neural activity delays the first appearance of coordinated movements in the embryos [29]. They found a sensitive period for this manipulation during late embryonic development, which overlaps with the critical period we observed for the manipulation of chos transmission. However, since the activity manipulation was applied to all neurons, the neurons responsible for the plastic change were not identified. And possible lasting effects of the manipulation later in larval life were not investigated, either. Our study complements and extends the work by Crisp et al. (2011) by showing that activity manipulation of specific sensory neurons during a critical developmental time window has lasting effects on the maturation of the motor circuits.

We found that two-hour perturbation of neural transmission by chos during a developmental window at 16–20 h AEL, but not at other embryonic and larval periods, has a lasting effect on the speed of locomotion. This critical time window corresponds to the developmental period when the circuits mature to generate locomotion with appropriate speed. Our results are thus consistent with the idea that sensory experience of the animals’ own movement, conveyed by chos, contributes to the maturation of the motor circuits. The critical period also corresponds to the period when sensory neurons and motor neurons elaborate axon terminals and dendrites, respectively, in the CNS [30]–[32]. Chos themselves are known to develop their axon terminals during this period [9], [23], [30]. Elaboration of the dendrites of motor neurons is known to occur in a manner that depends on presynaptic activity [31]. It is possible that final refinements of the synaptic connections of sensory neurons and interneurons similarly occur in an activity-dependent manner. The plastic change upon the manipulation of chos activity therefore likely occurs at the level of synaptic connection in sensorimotor circuits, as has been proposed for the sensory systems. Gross connectivity of chos as visualized with mCD8::GFP was normal upon the activity manipulation (Figure S1). Therefore, the plasticity may be mediated by fine-scale change(s) in the synaptic connections. Identifying the underlying structural and functional substrates for the observed behavioral plasticity is an important future research direction.

## Supporting Information

Figure S1 **No gross defects were seen in the connectivity of chos upon the activity manipulation.** Morphological analysis of the third instar larvae (96 h AEL; *iav-GAL4>UAS-Shi^ts^, UAS-mCD8::GFP*) with no temperature shift control (A) and with two-hour inhibition during embryonic stage (B, 17–19 h AEL). There were no gross defects in the projection and arborization of chos axons upon the activity manipulation (n = 4). Before the morphological analyses, behavioral analyses were performed to confirm the change in the speed of locomotion.
(TIF)

Movie S1 **Calcium imaging in chordotonal organs during muscle contractions.** Calcium imaging of chordotonal organs (chos) in dissected larva undergoing peristalsis (Quad-Speed, *iav-GAL4>UAS-GCaMP3*). Note that the activation of chos and segmental muscle contraction propagate at a similar timing. (9.5 MB MOV).
(MOV)
