# Supplementary figures and images for: Role of Sensory Experience in Functional Development of Drosophila Motor Circuits
Source: PLoS One. 2013 Apr 19;8(4):e62199. doi: 10.1371/journal.pone.0062199 (PMC3631234; doi:10.1371/journal.pone.0062199)

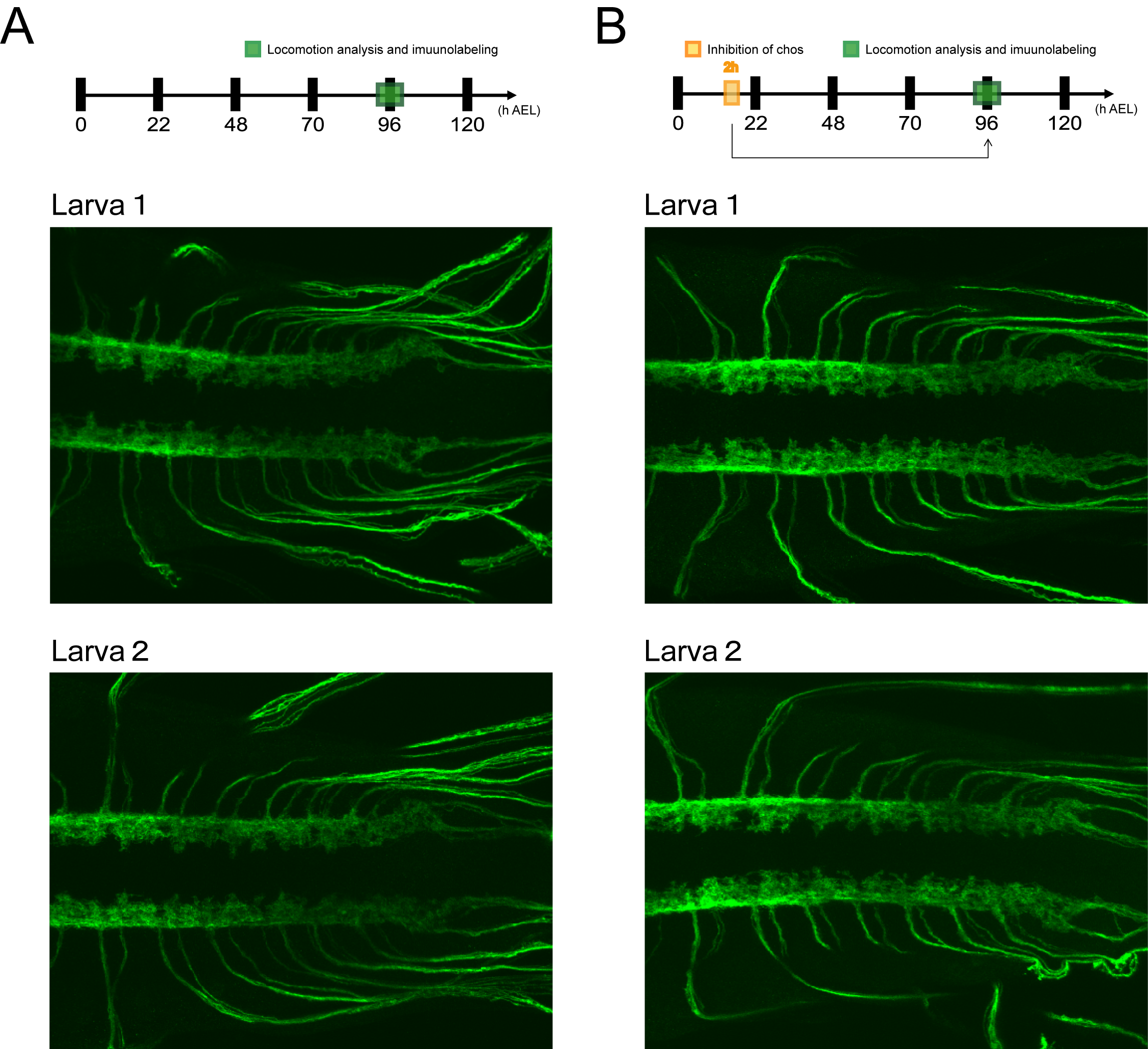

Supplement: Figure S1 — No gross defects were seen in the connectivity of chos upon the activity manipulation. Morphological analysis of the third instar larvae (96 h AEL; iav-GAL4>UAS-Shits, UAS-mCD8::GFP) with no temperature shift control (A) and with two-hour inhibition during embryonic stage (B, 17–19 h AEL). There were no gross defects in the projection and arborization of chos axons upon the activity manipulation (n = 4). Before the morphological analyses, behavioral analyses were performed to confirm the change in the speed of locomotion. (TIF) [file pone.0062199.s001.tif]
